# Axonal Varicosity Density as an Index of Local Neuronal Interactions

**DOI:** 10.1371/journal.pone.0022543

**Published:** 2011-07-21

**Authors:** Zi-Wei Zhang, Jun Il Kang, Elvire Vaucher

**Affiliations:** 1 School of Optometry, Université de Montréal, Montréal, Quebec, Canada; 2 Department of Physiology, Université de Montréal, Montréal, Quebec, Canada; Tokyo Medical and Dental University, Japan

## Abstract

Diffuse transmission is an important non-synaptic communication mode in the cerebral neocortex, in which neurotransmitters released from en passant varicosities interact with surrounding cells. In a previous study we have shown that the cholinergic axonal segments which were in the microproximity with dopaminergic fibers possessed a greater density of en passant varicosities compared to more distant segments, suggesting an activity-dependent level of en passant varicosities in the axonal zone of interaction. To further evaluate this plastic relationship, the density of cholinergic varicosities was quantified on fiber segments within the microproximity of activated or non-activated pyramidal cells of the prefrontal cortex (mPFC). Repetitive 14 days patterned visual stimulation paired with an electrical stimulation of the cholinergic fibers projecting to the mPFC from the HDB was performed to induce persistent axonal plastic changes. The c-Fos early gene immunoreactivity was used as a neuronal activity marker of layer V pyramidal cells, labelled with anti-glutamate transporter EAAC1. Cholinergic fibers were labeled with anti-ChAT (choline acetyltransferase) immunostaining. The density of ChAT+ varicosities on and the length of fiber segments within the 3 µm microproximity of c-Fos positive/negative pyramidal cells were evaluated on confocal images. More than 50% of the pyramidal cells in the mPFC were c-Fos immunoreactive. Density of ChAT+ varicosities was significantly increased within 3 µm vicinity of activated pyramidal cells (0.50±0.01 per µm of ChAT+ fiber length) compared to non-activated cells in this group (0.34±0.001; p≤0.05) or control rats (0.32±0.02; p≤0.05). Different types of stimulation (visual, HDB or visual/HDB) induced similar increase of the density of ChAT+ varicosities within microproximity of activated pyramidal cells. This study demonstrated at the subcellular level an activity-dependent enrichment of ChAT+ varicosities in the axonal zone of interaction with other neuronal elements.

## Introduction

The modulation of the cortical neuronal activity by the cholinergic system is mainly exerted via diffuse transmission by acetylcholine (ACh) released from varicosities along axons [1]–[8]. Synaptic transmission also occurs but more rarely. This diffuse transmission has been suggested in the medial prefrontal cortex (mPFC), where cholinergic neurons of the horizontal limb of the diagonal band of Broca (HDB) send long projections to its three subareas, the anterior cingulate (Cg1), the prelimbic (PrL) and the infralimbic (IL) cortex [9]–[13]. In these cortical regions, cholinergic stimulation induces modulation of pyramidal cells activity and synaptic plasticity [12]–[16], including neuroplasticity in layer V pyramidal neurons of mPFC [17], structural alteration of the pyramidal cells [18], glutamatergic transmission modulation [19]–[23]. Through this diffuse interaction with glutamatergic cells, the prefrontal cholinergic fibers play an active role in mPFC cognitive functions [19], [24]–[32].

Recently, it has been suggested that *en passant* boutons on axonal segments are dynamic structures and their density along the axons is not uniform [33]–[36]. Particularly, we have shown that the density of cholinergic and dopaminergic varicosities was significantly higher within fiber segments in reciprocal microproximity compared to more distant segments, suggesting variations in the density of varicosities could be a useful index for neuronal interaction [34]. In stationary Macky monkeys, frequent *en passant* boutons addition and elimination along collaterals were observed with *in vivo* two-photon imaging [35], as seen for dendritic spines plasticity [37]–[38]. Cell type-specific rearrangements of axonal boutons were also observed in the mouse barrel cortex *in vivo* [36].

In the present study, we hypothesized that varicosity density along the axon can be an index of localized neuronal interactions. Cholinergic varicosities on fiber segments within the vicinity of activated pyramidal cells of mPFC were examined in visually and HDB electrically stimulated rats using triple immunolabelling and confocal microscopy. The stimulation paradigm was designed to allow both post-synaptic (visual stimulation) and pre-synaptic (HDB stimulation) activation of pyramidal cells in the mPFC which corresponds to the mode of cholinergic system functioning, i.e. enhancement of afferent sensory inputs through Hebbs-like synaptic plasticity [14], [39]. HDB stimulation activates the cell soma of the cholinergic projections to the mPFC to modulate pyramidal cells [40]–[41], whereas signal induced by visual stimulation was expected to be modulated and processed by the neuronal elements in mPFC as the final step of the visual processing hierarchy [12], [41]. A two weeks repetitive stimulation was designed to allow structural changes correlating this consolidated neuronal network. Activation of the glutamatergic pyramidal cells was identified by a colocalization of the early gene c-Fos expression and the excitatory amino acid carrier protein 1 (EAAC1), a neuron-specific glutamate transporter (GluT) [34], [42]–[43]. Cholinergic fibers and varicosities were labelled by choline acetyltransferase (ChAT) [43]. Consistent with our hypothesis, significant elevation of ChAT+ varicosity density in the vicinity of c-Fos+/GluT(EAAC1)+ cells compared to those in the vicinity of c-Fos negative pyramidal cells were observed, suggesting dynamics of varicosity density as a useful index for the neuronal activity.

## Methods

### Animal preparation

Thirteen male Long Evans rats (300–325 g) obtained from Charles River Canada (St-Constant, Québec, Canada) were housed in a temperature-controlled room (21–25°C) under natural daylight, and had free access to food and water. All procedures were approved by the local Animal Care Committee (Comité de déontologie de l'expérimentation sur les animaux) and were conducted in accordance with the guidelines of the Canadian Council on Animal Care (protocol number: 10-133).

For chronic electrode HDB implantation, animals were anesthetized with isoflurane (induction 5%, maintain 3%) and placed in a stereotaxic apparatus. The stimulating tungsten electrode (impedance: 2 mΩ, FHC, Bowdoinham, ME, USA) denuded at each tip was implanted in the left HDB (mm from Bregma: AP −0.3, L +2.0, DV −9.0) via a hole made by a drill. The electrode was secured by dental cement. A post-operative delay of 2 days was allowed before any further experiment.

### Stimulation paradigm

Awake rats were restrained and head fixed in a frame surrounded by three monitors (one in the front and two at each side, placed 21 cm away from the eyes of the rat) during 10 min for 14 days. Three groups of rats were prepared for treatment. Control group (n = 4) received no visual stimulation (grey screen) and no HDB stimulation, visually stimulated group (n = 3) received visual stimulation (sine-wave grating) only, HDB stimulated group (n = 3) received HDB electrical stimulation only, and visual/HDB stimulated group (n = 3) received both HDB electrical stimulation and visual stimulation. All 13 animals including the controls received a chronic electrode implantation. Visual training was accomplished via exposition of the animal to a sine-wave grating stimulus (orientation 30°, contrast 90%, 0.12 cycle/deg, phase converting at 1 Hz, produced by Vpixx software, v 8.5; Sentinel Medical Research Corp., Quebec, Canada) displayed on the three computer monitors (LG, luminance 37 cd/m2) for 10 minutes. HDB electrical stimulation was performed over the same 10 min period using pulses (100 Hz, 0.5 ms, 50 µA, 1 sec on/1 sec off) generated (Pulsemaster A300, WPI, Sarasota, FL) and delivered through an isolation unit (WPI 365, WPI, Sarasota, FL) through the chronically implanted electrode.

### Immunocytochemistry

One week after the last stimulation, animals were anesthetised with isoflurane, and received a 20 min visual stimulation. This one week period was allowed for consolidation of the neuronal network involved during training and also for measuring discrimination performance of rats in the visual water maze task [43]–[45] (data outside of the focus of the present paper, not shown). Rats were then deeply anesthetised with pentobarbital (54 mg/kg body weight i.p.) and perfused transcardially with 4% paraformaldehyde at room temperature. After perfusion, brains were harvested, post fixed for 2 h in fresh fixative and stored in 0.1 M phosphate buffer (PBS, pH = 7.4) overnight. Serial coronal vibratome sections (35 µm thick) from mPFC (at the level of Bregma +2.7−2.2 mm) were cut using a vibratome (Leica microsystems), collected serially in 24 wells plates (4 sections/well) in 0.1 M phosphate buffer (PBS, pH = 7.4), so the AP level of the sections could be easily identified according to their position in the plate and anatomical references. Sections were then stored in antifreeze (30% glycerol, 30% ethylene glycol, 40% NaPB 0.24 M) solution at −20°C until further use. Selected sections were thoroughly washed in 0.1 M PBS and incubated in 0.3% H_2_O_2_ in 0.1 M PBS for 20 min. The sections were then washed and incubated in 1.5% donkey serum in PBS-T (0.1 M phosphate buffer, pH 7.4 and 0.25% triton x-100) for 1 h for non specific sites blockade. To examine the ChAT+ varicosities in vicinity of c-Fos expressing pyramidal cells, rat brain sections were incubated in a cocktail of primary antibodies for 24 h at room temperature (Table 1): anti-ChAT (1∶200; Chemicon, Billerica, MA, USA) for cholinergic fibers; anti-c-Fos (1∶10000; Oncogene Research Products, San Diego, CA, USA) for c-Fos expressing cells [43], [46]–[47] and anti-GluT(EAAC1) (1∶500, Chemicon, Billerica, MA, USA) for pyramidal cells [34], [42]–[43]. The morphology of GluT(EAAC1)+ cells was carefully examined and the analysis was made on the cells showing a clear pyramidal cell morphology. They were thus named pyramidal cells instead of GluT(EAAC1)+ cells. After washing, the sections were incubated in secondary antibodies conjugated with fluorophore FITC, CY5, TRITC (Jackson Immunoresearch, West Grove, PA, USA) for 2 h 30 (Table 1). To examine the c-Fos+ activity and the number of pyramidal cells on a larger scale, adjacent sections were double immunostained with anti-cFos and anti-GluT(EAAC1), and coupled with TRITC and FITC. Then the sections were washed and mounted onto slides with Vectashield mounting medium (Vector Laboratories, Burlingame, CA, USA).

**Table 1:** Combination of primary and secondary antibodies for double and triple-fluorescent staining procedures.

*Series*	Primary antibodies			Secondary antibodies	
	Antigen	Host	Dilution	Donkey IgG	Dilution
*ChAT/c-Fos/GluT(EAAC1)*	ChAT	goat	1∶200	Anti-goat-FITC	1∶200
	c-Fos	rabbit	1∶10000	Anti-rabbit-CY5	1∶200
	GluT(EAAC1)	mouse	1∶500	Anti-mouse-TRITC	1∶200
*c-Fos/GluT(EAAC1)*	c-Fos	rabbit	1∶10000	Anti-rabbit-CY5	1∶200
	GluT(EAAC1)	mouse	1∶1000	Anti-mouse-TRITC	1∶200

Abbreviations: ChAT: choline acetyltransferase; GluT(EAAC1): glutamate transporter.

### Confocal microscopy and quantification

Confocal microscopy offers a good means to explore into the neuroanatomical relationships in the three dimensions (x, y, z). Argon laser (excitation at 488 nm) was used for excitation of FITC, Helium Neon laser (excitation at 543 nm) was used for excitation of TRITC and Helium Neon laser (excitation at 633 nm) was used for excitation of CY5 via the Leica SP2 confocal microscope (Leica Microsystems, Wetzlar, Germany). The pinhole size was always set at Airy One (Beam expander 3) and the zoom factor remained at 1 throughout the imaging. On the sections with triple immunostaining, Z-series of images were taken using 100× oil lens (Plan Apochromatic; Numerical aperture: 1.4) in sequential scanning mode and with an image size of 1024×1024 pixels for each channel and captured by Leica Confocal Software (LCS lite). Sections with c-Fos/GluT(EAAC1) double immunostaining were imaged using 40× oil lens in sequential scanning mode and with an image size of 1024×1024 pixels. The step size between any two consecutive optical sections was set at 1 µm. A confocal projection was a series of horizontal optical scanning sections in the same image. When a projection was generated, the sampling points of the individual images superimposed along the projection axis were examined throughout all optical sections. In a maximum projection, the maximum intensity value was displayed. On those coronal sections, the dendrites of the pyramidal cells in layer V extended toward the direction of the pial surface and sometimes spanned into upper layers. The field size of 150 µm×150 µm was sufficient to capture the complete shape of pyramidal soma and dendrites in the XY direction. To fully capture the full shape of soma and dendrites of pyramidal cells in the Z-direction, projection images with a total depth of 3 µm instead of single optical scanning images were used for quantification. Two ROI's (Regions of Interest) were randomly chosen within each subregion of the mPFC.

Based on functional or anatomical relationship between the mPFC and BF [9], [12]–[13], Cg1, PrL and IL were analyzed. The neuronal relationships within layer V were quantified because they constitute the principal cortical output layer [18], [48]. Confocal images could be confidently ascribed to layer V on the basis of the depth of the image below the surface and the characteristics of the distribution and shape of pyramidal cells. The density of ChAT+ varicosities on fiber segments within the microproximity (3 µm or less) [34] of pyramidal cells immunoreactive or not with c-Fos in visual/HDB animals were quantified on projection images using Leica confocal software (LAS AF lite). In contrast, only the density of ChAT+ varicosities on fiber segments within the microproximity c-Fos negative pyramidal cells were examined in control animals, since 98% of the pyramidal cells were c-Fos negative (Table 2). To see if it is possible to further differentiate the major factor(s) contributing to the morphological changes of cholinergic fibers associated with local neuronal activity, the density of ChAT+ varicosities on fiber segments within the microproximity c-Fos positive pyramidal cells in HDB stimulation only group and in visual stimulation only group were also examined in layer V of IL. Considering the rapid hydrolysis of ACh by acetyl cholinesterase [3]–[4], [6], the present research adopted a conservative within-limit number for efficient diffuse transmission of ACh, 3 µm in three-dimensions (x, y, z axis in confocal microscopy) [34], as a maximal sphere of concentration for ACh to induce significant effect [49]–[50]. The varicosity density didn't vary much with different sections or animals within the same group, indicating accurate and reliable detection of varicosities. In total, ChAT+ varicosities were counted on 4931.88 µm of ChAT+ fiber segments within the vicinity of 546 pyramidal cells (259 of which being c-Fos+). The density of c-Fos+, pyramidal and c-Fos+/pyramidal cells were quantified by visual examination using photoshop CS3. Each ROI (i.e, each single projection image) covered 3 µm in depth of the tissue and corresponded to a field size of 4.3×10^5^ µm^3^. Therefore, the number of c-Fos+ cells was expressed as the number per 4.3×10^5^ µm^3^. In total, 3764 pyramidal cells and 4012 c-Fos+ cells were quantified on confocal images of double immunolabeling of c-Fos+ and pyramidal cells (40×, oil), within a total sampling area of 5.20×10^7^ µm^3^.

**Table 2:** Number of c-Fos+ cells and Pyramidal cells in layer V of mPFC.

	Number of c-Fos+ cells	Number of Pyramidal cells	Percentage of Pyramidal cells which are c-Fos+	Percentage of c-Fos+ cells which are GluT(EAAC1)+
***Visual/HDB stimulation***				
Infralimbic Ctx	87±39& ▴	30±2▴	73&	25▴
Prelimbic Ctx	80±34& ▴	32±12	78& ▴	31▴
Cingulate Ctx	53±27&	31±5	65&	38& ▴
***Visual stimulation***				
Infralimbic Ctx	13±6▪	39±15	9* ▪	33▪
Prelibic Ctx	8±6* ▪	33±14	5▪	23▪
Cingulate Ctx	7±6▪	30±5	8* ▪	51*
***HDB stimulation***				
Infralimbic Ctx	39±15▴ ▪ ▵	46±8▴	49▪ ▵	59▴ ▪ ▵
Prelimbic Ctx	37±15▴ ▪ ▵	42±14	48▴ ▪ ▵	60▴ ▪ ▵
Cingulate Ctx	22±11▪ ▵	38±5	35▪ ▵	62▴ ▵
***Control***				
Infralimbic Ctx	5±3& ▵	38±13	2* & ▵	16▵
Prelimbic Ctx	2±1* & ▵	37±8	2& ▵	24▵
Cingulate Ctx	1±1& ▵	38±11	0* & ▵	0* ▵ &

All values are means ± SEM (number of cells/4.3×10^5^ µm^3^) or percentage of colocalization of Pyramidal and c-Fos+ cells from 13 adult rats in visual/HDB stimulation group. Abbreviations: Ctx: cortex; GluT(EAAC1): glutamate transporter; HDB: horizontal limb of the diagonal band; mPFC: medial prefrontal cortex.

*p≤0.05, Mann-Whitney U test, two-tailed, group-specific difference between control and visual stimulation.

▵ p≤0.05, Mann-Whitney U test, two-tailed, group-specific difference between control and HDB stimulation.

& p≤0.05, Mann-Whitney U test, two-tailed, group-specific difference between control and visual/HDB stimulation.

▴ p≤0.05, Mann-Whitney U test, two-tailed, group-specific difference between visual/HDB stimulation and HDB stimulation.

▪ p≤0.05, Mann-Whitney U test, two-tailed, group-specific difference between visual stimulation and HDB stimulation.

### Statistics

All the statistics in the present study were processed with SPSS (17.0). For each biological parameter investigated in this study (varicosity density, varicosity diameter, fiber segment length, c-Fos activity, percentage of c-Fos activated pyramidal cells, etc), region-specific comparisons were carried out between any of the subregions in the mPFC in each group (Wilcoxon signed-rank test for paired samples, p≤0.05, two-tailed), and group-specific comparisons were made between any of the groups in each subregion of the mPFC (Mann-Whitney U test, p≤0.05, two-tailed).

## Results

### C-Fos activation of GluT (EAAC1)+ cells in layer V of the mPFC

**Figure 1:**
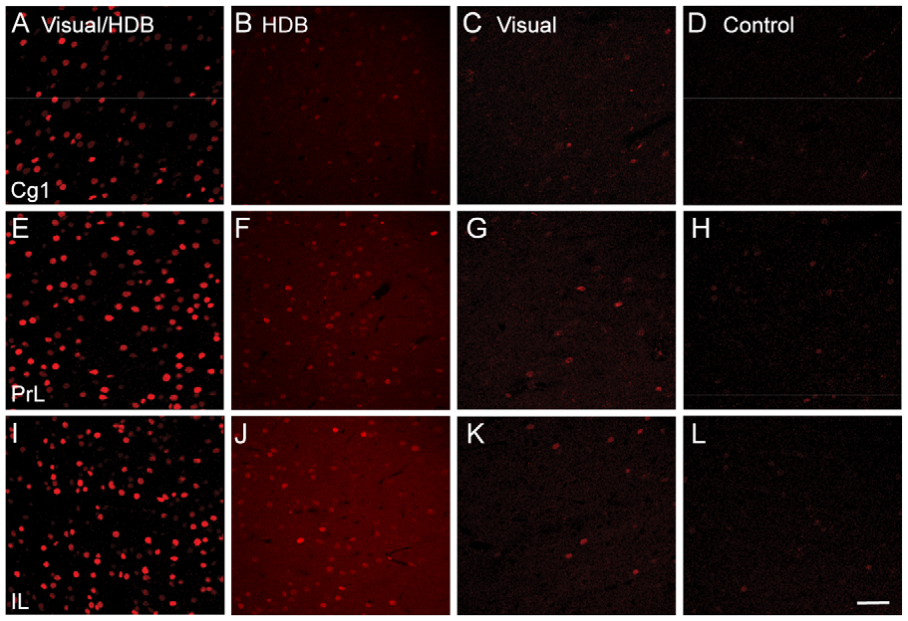
c-Fos immunoreactivity in layer V of the mPFC. Representative examples of c-Fos activity in layer V of Cg1 (A–C), PrL (D–F), and IL (G–I) in visual/HDB stimulated (A,D,G), visual stimulated (B,E,H) and control (C,F,I) rats. c-Fos+ cells appeared as red cores. In the visual/HDB stimulation group, there were significantly lower c-Fos+ cells in Cg1 compared to PrL or IL. There was no significant difference in c-Fos activity between PrL and IL. In all the three subregions of mPFC there was significant enhancement of c-Fos activity in visual/HDB stimulation rats compared to control or visual stimulated rats. Abbreviations: Cg1: cingulate cortex; IL: infralimbic cortex; mPFC: medial prefrontal cortex; PrL: prelimbic cortex. Scale bar: 50 µm.

c-Fos immunoreactivity in layer V of the mPFC was significantly greater in visual/HDB stimulation and HDB stimulation group compared to control or to visual stimulation groups (Mann-Whitney Test, p≤0.05, two-tailed, Fig. 1, Table 2, 3). It was also significantly greater in PrL and IL of visual/HDB stimulation group compared to HDB stimulation group but not in Cg1 (Table 2), suggesting that our site of HDB stimulation exerted a modulating effect on sensory processing mainly in PrL and IL instead of Cg1. c-Fos immunoreactivity in PrL was also significantly greater in visually stimulated rats compared to control rats (Mann-Whitney Test, p≤0.05, Fig. 1, Table 2, 3). There was no regional difference in the number of c-Fos+ cells (Wilcoxon signed-rank test, p>0.05, two-tailed; Fig. 1, Table 2, 3).

**Table 3:** *p* values of group-specific effects.

	Control vs. Visual	Control vs. HDB	Control vs. Visual/HDB (cFos+)	Control vs. Visual/HDB (cFos−)	Visual vs. HDB	Visual vs. Visual/HDB (cFos+)	HDB vs. Visual/HDB (cFos+)	Visual/HDB (cFos+) vs. Visual/HDB (cFos−)
***Number of cFos+ cells***								
Cg1	0.077	0.034*	0.034*	—	0.050*	0.050*	0.127	—
PrL	0.034*	0.034*	0.034*	—	0.050*	0.050*	0.050*	—
IL	0.077	0.034*	0.034*	—	0.050*	0.050*	0.050*	—
***Number of pyramidal cells***								
Cg1	0.480	1.000	0.480	—	0.127	0.513	0.127	—
PrL	0.724	0.724	0.724	—	0.827	0.827	0.275	—
IL	0.724	0.480	0.289	—	0.376	0.513	0.050*	—
***ChAT+ varicosity density***								
Cg1	—	—	0.034*	0.480	—	—	—	0.050*
PrL	—	—	0.034*	1.000	—	—	—	0.050*
IL	0.032*	0.032*	0.034*	0.157	1.000	1.000	0.275	0.050*
***ChAT+ varicosity diameter***								
Cg1	—	—	0.724	0.289	—	—	—	0.513
PrL	—	—	0.077	0.289	—	—	—	0.827
IL	0.289	0.724	0.034*	0.034*	0.513	0.050*	0.513	0.513
***ChAT+ fiber length***								
Cg1	—	—	1.000	0.034*	—	—	—	0.127
PrL	—	—	0.289	0.034*	—	—	—	0.275
IL	0.034*	0.0513	0.034*	0.034*	0.513	0.275	0.827	0.275

All values are obtained *p* values of group-specific effects in the c-Fos immunoreactivity and ChAT+ varicosity density/diameter and ChAT+ fiber length within microproximity of pyramidal cells in 4 groups of rats: control, visual stimulation only, HDB stimulation only, and visual/HDB stimulation. Symbol* indicates significant *p* values (Mann-Whitney U test, p≤0.05, two-tailed).

**Figure 2:**
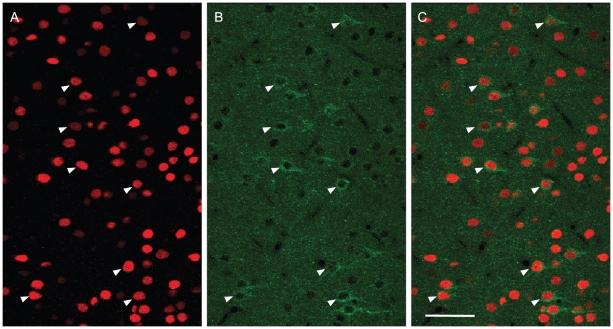
Colocalization of c-Fos and GluT(EAAC1) in pyramidal cells in layer V of mPFC. Representative examples of c-Fos (red, A) and GluT(EAAC1) (green, B) immunostaining and their colocalization (C) in layer V of PrL in the mPFC of visual/HDB stimulated rats. Arrowheads point to examples of colocalization of c-Fos+ cells and pyramidal cells. Note that the majority of pyramidal cells were immunoreactive for c-Fos, whereas only a small proportion of c-Fos+ cells were stained for GluT(EAAC1). Abbreviations: ChAT: choline acetyltransferase; GluT(EAAC1): glutamate transporter; mPFC: medial prefrontal cortex; PrL: prelimbic cortex. Scale bar: 50 µm.

Consequently, the proportion of c-Fos immunoreactive pyramidal cells was significantly different (Mann-Whitney Test, p≤0.05, Table 2, 3) among the four groups of rats. In visual/HDB stimulation group, the majority of the pyramidal cells were c-Fos immunoreactive (65% in Cg1, 78% in PrL, and 73% in IL) (Fig. 2, Table 2, 3). This proportion was significantly lower in HDB stimulation group (35% in Cg1, 48% in PrL and 49% in IL). Only 0 to 2% and 5 to 9% of the pyramidal cells were c-Fos immunoreactive in the mPFC from control rats and visually stimulated rats, respectively. This was significantly lower compared to either visual/HDB stimulation group (Mann-Whitney Test, p≤0.05, Table 2, 3) or HDB stimulation group (Mann-Whitney Test, p≤0.05, Table 2, 3), suggesting the frontal cortical representation of visual stimuli was enhanced by HDB input into mPFC. No significant difference in the number of pyramidal cells in mPFC was found among the four different groups of animals (Table 2), with the exception of IL between HDB stimulation group and visual/HDB stimulation group, which could be possibly attributed to a slight overexpression of GluT(EAAC1) protein in the HDB stimulated animals – resulting in an enhanced detectability of the glutamatergic cells. No significant regional difference in the proportion of c-Fos+ pyramidal cells was found for each group (Wilcoxon signed-rank test, p>0.05, two-tailed).

The proportion of c-Fos+ cells that were glutamatergic (labelled for GluT(EAAC1)) was significantly greater in HDB stimulation group (from 59 to 62%) compared to visual/HDB stimulation groups (from 25 to 38%, Mann-Whitney Test, p≤0.05, Table 2), or visual stimulation group (from 23 to 33%, Mann-Whitney Test, p≤0.05, Table 2).

### Activation-dependent elevation of the density of ChAT+ varicosities in layer V of the mPFC

**Figure 3:**
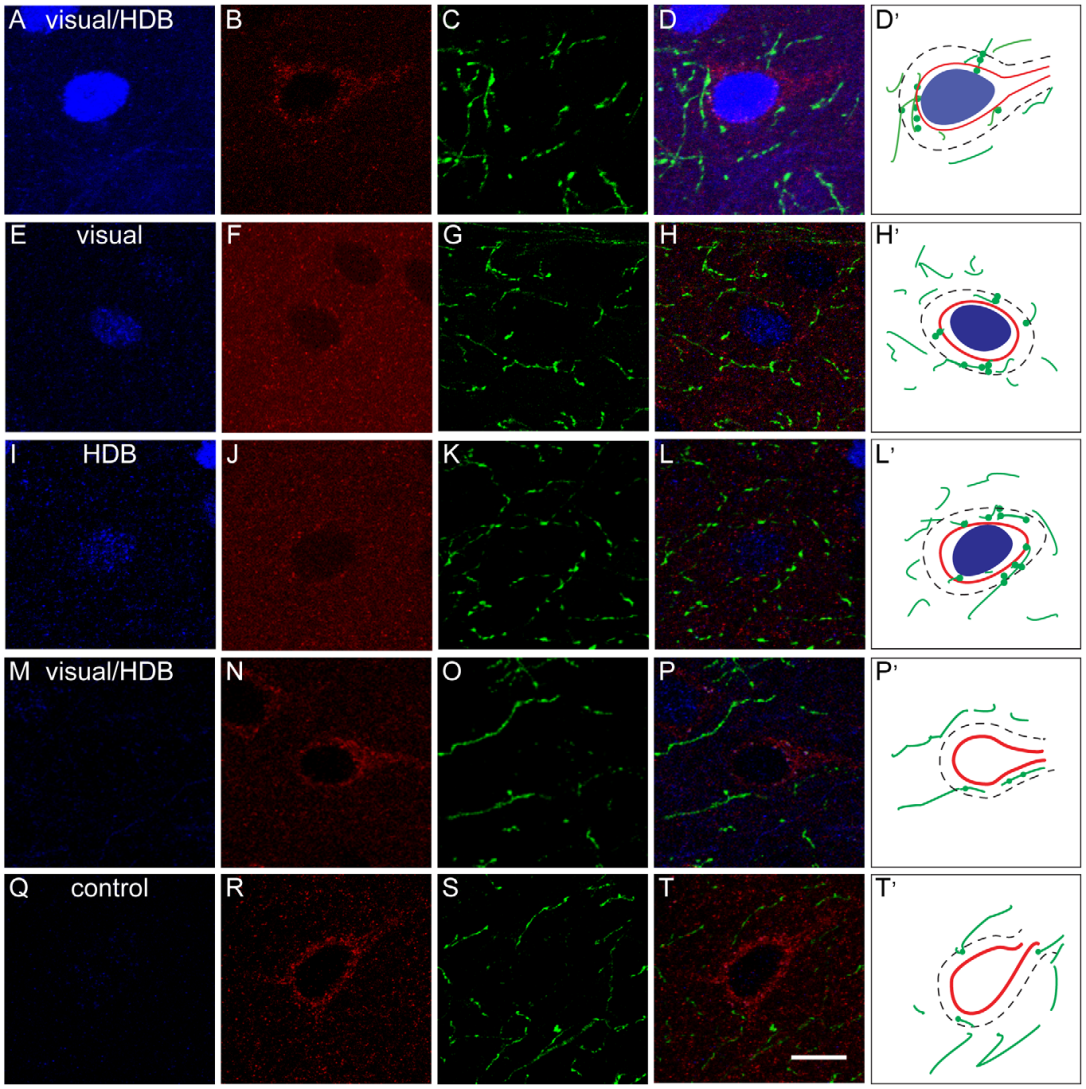
Evaluation of ChAT+ varicosity density on fiber segments within microproximity of activated or nonactivated pyramidal cells. Representative examples of projection images of triple immunostaining for c-Fos (blue), GluT(EAAC1) (red) and ChAT (green) in layer V of the mPFC (A–P) of ChAT+ varicosity in microproximity of activated pyramidal cells in visual/HDB stimulation group, visual stimulation only group, and HDB stimulation only group (the three top panels), and for ChAT+ varicosity in microproximity of nonactivated pyramidal cells in the visual/HDB stimulation group and the control group (the bottom two panels) D',H',L',P', T': Drawing of ChAT+ fibers (green lines) within 3 µm microproximity (dashed line) of c-Fos+ pyramidal cells (red perimeter with a blue nucleus) or non-activated pyramidal cells (red perimeter without blue nucleus) in layer V of the mPFC. Representative images were taken from IL and PrL. ChAT+ varicosities (illustrated by dots on the green ChAT+ fibers) were quantified on fiber segments within 3 µm microproximity of pyramidal cells. Note that an increased density of varicosities was seen in segments in microproximity of activated cells compared to non-activated cells. Abbreviations: ChAT: choline acetyltransferase; GluT(EAAC1): glutamate transporter; Cg1: cingulate cortex; IL: infralimbic cortex; mPFC: medial prefrontal cortex; PrL: prelimbic cortex. Scale bar: 10 µm.

**Figure 4:**
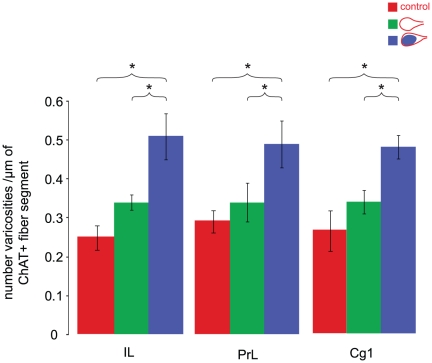
ChAT+ varicosities density on fiber segments within microproximity of activated or nonactivated pyramidal cells. Within the visual/HDB stimulation group, the density of ChAT+ varicosities on fiber segments within microproximity of nonactivated cells (green bars) was lower than on fiber segments within microproximity of activated pyramidal cells (blue bars) in layer V of every subregion of mPFC investigated. In contrast, the density of ChAT+ varicosities fiber segments within microproximity of nonactivated cells in the control group (red bars) was not significantly different from those within microproximity of nonactivated cells in the visual/HDB stimulation group, but significantly lower than those within microproximity of activated pyramidal cells in the visual/HDB stimulation. Symbol * indicate significant difference in the density of ChAT+ varicosities on fiber segments within microproximity of activated pyramidal cells compared to those within microproximity of nonactivated pyramidal cells (p≤0.05, Mann-Whitney U test, two-tailed). Abbreviations: Cg1: cingulate cortex; ChAT: choline acetyltransferase; IL: infralimbic cortex; PrL: prelimbic cortex.

In layer V of the mPFC of visual/HDB stimulation group, a higher density of ChAT+ varicosities was found on axonal fiber segments within the vicinity (3 µm or less) of c-Fos+ pyramidal cells compared to those within the vicinity of non activated pyramidal cells in visual/HDB stimulation or control group, suggesting a localized activity-dependent enrichment of ChAT+ varicosities (Mann-Whitney Test, p≤0.05, two-tailed, Fig. 3, 4, Table 3, 4). No significant difference was found in the ChAT+ varicosity density within the proximity of non-activated pyramidal cells between visual/HDB stimulation group and control group in all three subareas (Mann-Whitney Test, p = 0.034, two-tailed, Table 3, 4, Fig. 4), indicating the increase of ChAT+ varicosity density within the proximity of c-Fos+ pyramidal cells in visual/HDB stimulation group was indeed due to relevant neuronal activity. In the control group, all the quantification was done on c-Fos negative pyramidal cells, since 98% of the pyramidal cells were c-Fos negative (Table 2).

**Table 4:** Density and diameter of ChAT+ varicosities and length of ChAT+ fiber segments within microproximity of Pyramidal cells in Layer V of mPFC.

	IL	PrL	Cg1
***Visual/HDB stimulation***			
*Microproximity with c-Fos+ pyramidal cells*			
Density of ChAT+ varicosities	0.51±0.06* &	0.49±0.06* &	0.48±0.03* &
Diameter of ChAT+ varicosities(nm)	608±10&	602±14	601±28
ChAT+ fiber length(µm)	9.9±1.1	9.7±1.5	9.4±2.2*
*Microproximity with non-activated pyramidal cells*			
Density of ChAT+ varicosities	0.34±0.02*	0.34±0.05*	0.34±0.03*
Diameter of ChAT+ varicosities(nm)	626±36▴	598±24	608±20
ChAT+ fiber length(µm)	9.1±0.9▴	8.3±0.6▴	7.1±0.4▴
***Control***			
*Microproximity with non-activated pyramidal cells*			
Density of ChAT+ varicosities	0.31±0.03& + ‡	0.34±0.02&	0.32±0.04&
Diameter of ChAT+ varicosities(nm)	581±6& ▴	580±16	596±15
ChAT+ fiber length(µm)	12.9±1.1▴ +	10.9±0.9▴	10.1±0.7▴

All values are means ± SEM in visual/HDB stimulation rats and control rats. Density of ChAT+ varicosities is expressed in number of varicosities/µm of ChAT+ fiber segment. Diameter of ChAT+ varicosities is expressed in nm. ChAT+ fiber length is expressed in µm. Abbreviations: Cg1: cingulate cortex; ChAT: choline acetyltransferase; IL: infralimbic cortex; PrL: prelimbic cortex; GluT(EAAC1):glutamate transporter; HDB: horizontal limb of the diagonal band; mPFC: medial prefrontal cortex.

*p≤0.05, Mann-Whitney U test, two-tailed, comparing microproximity of Pyramidal/c-Fos+ cells to microproximity of Pyramidal cells not immunoreactive to c-Fos within visual/HDB stimulation group.

& p≤0.05, Mann-Whitney U test, two-tailed, comparing microproximity of Pyramidal/c-Fos+ cells in visual/HDB stimulation group to microproximity of Pyramidal cells not immunoreactive to c-Fos in control group.

▴ p≤0.05, Mann-Whitney U test, two-tailed, comparing microproximity of Pyramidal cells not immunoreactive to c-Fos in visual/HDB stimulation group to control group.

+ p≤0.05, Mann-Whitney U test, two-tailed, comparing microproximity of Pyramidal/c-Fos+ cells in visual stimulation group to microproximity of Pyramidal cells not immunoreactive to c-Fos in control group.

‡ p≤0.05, Mann-Whitney U test, two-tailed, comparing microproximity of Pyramidal/c-Fos+ cells in HDB stimulation group to microproximity of Pyramidal cells not immunoreactive to c-Fos in control group.

In layer V of IL a higher density of ChAT+ varicosities was found on axonal fiber segments within the vicinity of c-Fos+ pyramidal cells of both visual stimulation group (0.47±0.03 per µm of ChAT+ fiber length) and HDB stimulation group (0.45±0.04 per µm of ChAT+ fiber length) compared to those within the vicinity of c-Fos negative pyramidal cells in control group (0.31±0.03 per µm of ChAT+ fiber length, Mann-Whitney Test, p = 0.032, two-tailed, Fig. 3, Table 3, 4), suggesting either visual stimulation alone or HDB stimulation alone could induce localized activity-dependent enrichment of ChAT+ varicosities in the mPFC. The increase of the ChAT+ varicosity density within the microproximity of c-Fos+ pyramidal cells was about the same level in these groups as in visual/HDB stimulation group, since no statistical difference was found between these groups (Mann-Whitney Test, p>0.05, two-tailed, Fig. 3, Table 3, 5).

**Table 5:** Density and diameter of ChAT+ varicosities and length of ChAT+ fiber segments within microproximity of activated pyramidal cells in Layer V of IL.

IL	Visual stimulation	HDB stimulation	Visual/HDB stimulation
*Microproximity with c-Fos+ pyramidal cells*			
Density of ChAT+ varicosities	0.47±0.03*	0.45±0.04*	0.51±0.06*
Diameter of ChAT + varicosities (nm)	578±21	598±27	608±10*
ChAT+ fiber length (µm)	9.3±1.6*	10.2±1.4	9.9±1.1

All values are means ± SEM in 3 groups of rats: visual stimulation only, HDB stimulation only, and visual/HDB stimulation. No statistical significance was found among these 3 groups regarding ChAT+ varicosity density, diameter or fiber length (p>0.05, Mann-Whitney U test, two-tailed). Density of ChAT+ varicosities is expressed in number of varicosities/µm of ChAT+ fiber segment. Diameter of ChAT+ varicosities is expressed in nm. ChAT+ fiber length is expressed in µm. Abbreviations: ChAT: choline acetyltransferase; IL: infralimbic cortex; HDB: horizontal limb of the diagonal band.

*p≤0.05, Mann-Whitney U test, two-tailed, comparing microproximity of Pyramidal/c-Fos+ cells to microproximity of Pyramidal cells not immunoreactive to c-Fos in control group.

### Diameter of ChAT+ varicosities in layer V of the mPFC

The diameter of ChAT+ varicosities within the vicinity of c-Fos+ pyramidal cells or pyramidal cells of IL in visual/HDB stimulation group were significantly bigger than those within the vicinity of pyramidal cells of IL in control group and in visual group, which could suggest more modulation from HDB in IL compared to PrL and Cg1 (Mann-Whitney Test, p≤0.05, two-tailed, Table 3, 4).

No regional difference among Cg1, PrL, and IL was found for each parameter (Wilcoxon signed-rank test, p>0.05, two-tailed).

### Longer ChAT+ fiber segment within microproximity of c-Fos+ pyramidal cells in layer V of Cg1

ChAT+ fiber segments within the microproximity of c-Fos+ pyramidal cells in control group were significantly longer than those within the microproximity of c-Fos activated pyramidal cells in Layer V of IL in visual/HDB stimulation group (Mann-Whitney Test, p = 0.034, two-tailed, Table 3, 4), indicating that enhanced varicosity density within the vicinity of c-Fos activated pyramidal cells was not due to longer fiber length. ChAT+ fiber segments within the microproximity of pyramidal cells in mPFC in control animals were significantly longer than those within the microproximity of pyramidal cells in visual/HDB stimulation group (Mann-Whitney Test, p = 0.034, two-tailed, Table 3, 4).

No significant regional difference was found among the three subareas regarding ChAT+ fiber length in the vicinity of pyramidal cells in visual/HDB stimulation group (Wilcoxon signed-rank test, p>0.05, two-tailed).

## Discussion

The primary interest of the current study was to verify whether the density of axonal varicosities in a specific axonal segment was related to the level of local neuronal activity. To this purpose, segments of ChAT+ fibers stimulated repetitively were analyzed in relation to their vicinity to mPFC pyramidal cells expressing or not the early gene c-Fos. The results show different level of modulation of mPFC pyramidal cells by visual and HDB cholinergic inputs. Significant elevation of the density of ChAT+ varicosities within the microproximity of c-Fos+ pyramidal cells compared to those within the microproximity of nonactivated cells was demonstrated. This suggests dynamics of the varicosities along the axon relatively to local neuronal interaction.

### Activity-dependent modulation of pyramidal cells by HDB input in layer V of mPFC

5 to 9% of the pyramidal cells were c-Fos immunoreactive in the mPFC of visually stimulated rats which was significantly more than in control groups. They were significantly lower when compared either with visual/HDB stimulation group (70%) or HDB stimulation alone (50%) group, suggesting the strong c-Fos activation in the mPFC cortex was mainly due to HDB stimulation. The c-Fos activation of the pyramidal cells in the mPFC by visual stimulation alone was most probably due to post-synaptic activation brought upon by visual sensory input into the PFC from associational sensory cortices [51]–[53], as already reported [54]–[56]. The strong c-Fos+ immunoreactivity elicited by HDB stimulation, most likely resulted from direct HDB projections to the mPFC cells and local fibers [57]. Such strong c-Fos activation after acute basal forebrain stimulation has already been reported in fronto-parietal cortex of rats [58]. Moreover, the pairing of visual and HDB stimulation induced significantly higher c-Fos immunoreactivity in mPFC than either visual stimulation alone or HDB stimulation alone, suggesting a potentiation of mPFC pyramidal cells response to sensory stimuli by cholinergic projections, as shown in other cortical areas [54]–[55]. Thus, the c-Fos activation of pyramidal cells in the mPFC in visual/HDB stimulation rats represented a combination of both sensory and cholinergic activation, with HDB stimulation contributing more than visual stimulation itself. The frontal cortical representation of visual stimuli was thus primarily enhanced by HDB input into mPFC [40], [43], both of which were suggested as part of cognitive mechanism [59]–[60]. Our results were consistent with the assumed role of cortical projecting cholinergic system from basal forebrain in facilitating extrinsic sensory stimuli [24], [61]–[62]. Visual stimulation paired with HDB stimulation enhanced c-Fos+ activity on a large scale, indicating the potential role of HDB as an important relay station in enhancing frontal cortical representation of visual stimuli, consistent with basal forebrain's role in enhancement of cognitive functions [29], [63]–[64].

In HDB stimulation group, about 60% of c-Fos+ cells were glutamatergic. It has been found that most pyramidal cells (78–90%) in layer V of the mPFC were within the proximity of cholinergic fibers [34], suggesting a conjoint activation of the pyramidal cells by sensory or associative afferents and cholinergic projections. The majority (65–78%) of the pyramidal cells of layer V were c-Fos+ in visual/HDB stimulated rats, consistent with the well-documented importance of pyramidal cells in cognition in the frontal cortex [65]–[69]. Our study was focussed on the cholinergic input from the HDB, but it should be noted that GABAergic or glutamatergic projections from the HDB [13] could also have contributed to the c-Fos signal in mPFC. As well, only a moderate proportion of c-Fos+ cells (40% in Cg1, 32% in PrL, and 29% in IL) were pyramidal cells in visual/HDB stimulation group, which might suggest the activation of other phenotypes of intracortical neurons [70].

### Dynamics of varicosities as an index of localized neuronal activity

The density of ChAT+ varicosities within the vicinity of c-Fos+ activated pyramidal cells in visual/HDB stimulation group was significantly elevated compared to those within the vicinity of non-activated pyramidal cells either in visual/HDB stimulation group or in control group, a piece of evidence supporting the modulation of the pyramidal cell activity by HDB projections into the mPFC, and thus varicosity density being a good index for local neuronal activity. Consistently, no significant difference was found in the ChAT+ varicosity density within the microproximity of non-activated pyramidal cells between visual/HDB stimulation group and control group, indicating the increase of varicosity density within the microproximity of activated pyramidal cells in visual/HDB stimulation group was indeed due to relevant neuronal activity. Dendritic arbors of the pyramidal cell could not be visualized with our GluT(EAAC1) antibody, but it might be possible that this microproximity relationship includes axo-dendritic interactions since dendrites are filled of cholinergic receptors. It is probable that the c-Fos expression by pyramidal cells was induced by ACh, as c-Fos is not expressed in cholinergic lesioned animals in visual cortex [43]. However, as the c-Fos expression was maintained even one week after the last HDB stimulation, we suggest that there was also some backward crosstalk from the pyramidal cell to the cholinergic fibers. This crosstalk could involve local factors released from activated mPFC neurons, such as potassium, glutamate, neurotrophins which could maintain the state of activation of the cholinergic fibers. This is complementary to previous results suggesting that local neurons are able to induce ACh release from the cholinergic fiber terminals [57], [71]. Also, recurrent collaterals from the pyramidal cells might influence local cholinergic fibers. Stimulation paradigm is another factor which could affect the formation of new varicosities, such as the type, intensity and duration of the stimuli [72]–[74], as well as the age of the animals [73].

In order to elucidate whether the change in ChAT+ varicosity density was due to local factors or intrinsic axonal properties, the enrichment of ChAT+ varicosities in microproximity of c-Fos+ pyramidal cells in IL was quantified in visual stimulation alone or HDB stimulation alone conditions. In both cases, the density of ChAT varicosities was equivalent to the one in the visual/HDB group. This suggests both types of stimulation could contribute to the dynamics of ChAT+ varicosities and thus varicosities dynamics were due to interaction with local neurons rather than activation of the fiber *per se*. This is consistent with the fact that activation of HDB fibers did not result in increased varicosity density in microproximity to the non-activated pyramidal cells. Thus, it seems that different types of stimuli could induce the same level of varicosity enrichment, which should be investigated further.

**Figure 5:**
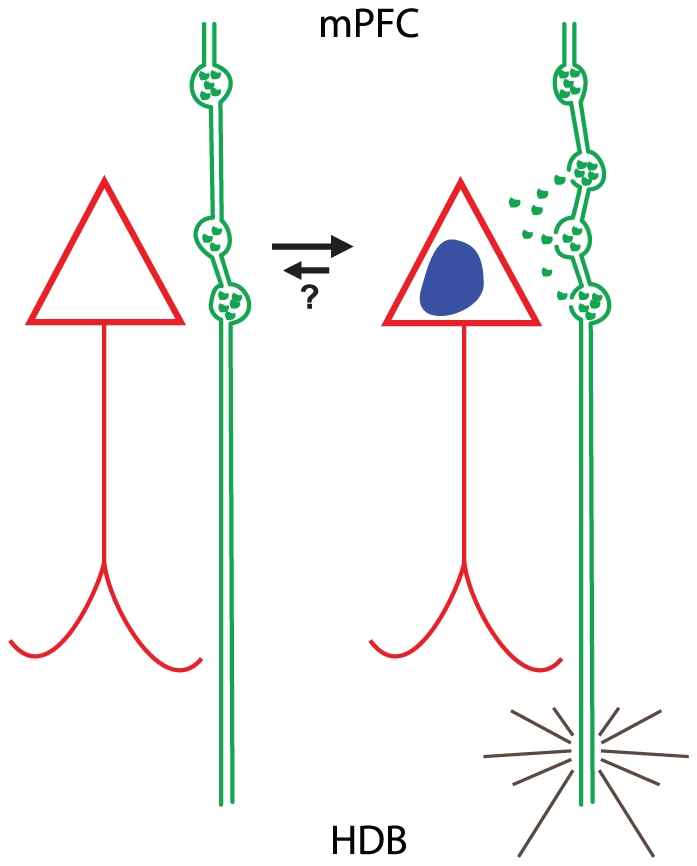
Schematic illustration of hypothetic duplication of the *en passant* varicosities due to repetitive neuronal activation. Repetitive activation (grey lines) of the cholinergic neurons in HDB could lead to induction of c-Fos (blue core) activation of pyramidal cells (red triangle) in mPFC and duplication of the cholinergic varicosities on their projecting fibers in vicinity of activated pyramidal cells. Whether this gain of varicosities is reversible remains to be determined (question mark). Abbreviations: HDB: horizontal limb of the diagonal band; mPFC: medial prefrontal cortex.

Our previous findings demonstrated significant elevation of ChAT+ and tyrosine hydroxylase+ varicosities on fiber segments within reciprocal microproximity of each other in the mPFC of normal adult rats [34], sustaining ACh/DA interaction evidences in this cortex. The present study verified that dynamics of ChAT+ varicosity density was related to the level of activity of pyramidal cells in the mPFC at a subcellular level, suggesting that the density of varicosity can be used as an index for local neuronal interaction (Fig. 5). Such plastic structural changes are extensively described in the dendritic arbor of pyramidal cells, where the dynamic and number of dendritic spines is modulated by local neuronal activity. In case of dendritic spines, repetitive activation of the synapses reinforces the strength of post and presynaptic elements and lead to duplication of the synapse [66], [75]–[76] to favor improved postsynaptic activation.

As well, recent studies suggest that varicosities spacing patterns seems regulated according to synaptic plasticity [35], [77] although some other studies propose that varicosities on axons from cortical neurons are formed and spaced in a regulated manner [78]. Varicosities on CA3-CA1 axonal shafts were found to be spaced non-uniformly along the axons. On short scales, varicosity spacing was found to be highly variable but not random [77], [79]. As well, synaptic boutons gain and loss were observed in adult visual cortex [35]. The higher density of ChAT+ varicosities on fiber segments innervating c-Fos+ activated pyramidal cells corroborate these previous hypotheses and link the density of varicosities to neuronal activity. Appearing of new *en passant* varicosities might be either due to formation de novo or mobilization of previously existing ones to new sites as fast as 1–2 h after stimulation as shown for presynaptic axonal terminals [80], [81], and may be correlated to the turnover of other neuronal elements in the same microenvironment [82]. We suggest that duplication of the *en passant* varicosities could happen in repetitive neuronal activation (Fig. 5). Whether this gain of varicosities is reversible remains to be determined, but previous studies tend to suggest it [35]. The length of the fiber segment sharing the microproximity with the activated cells was elevated in Cg1 of visually/HDB stimulated animals, suggesting that the surface of interaction between the fiber and the pyramidal cell increases after repetitive interaction. This increase of surface interaction and number of varicosity would result in increased ACh diffusion and putatively increased cholinergic transmission efficiency, which is consistent with our previous findings of more appositions between cholinergic and dopaminergic fibers in Cg1 despite its smallest fiber density compared to PrL and IL of mPFC [34]. In contrast, the diameter of the ChAT+ varicosities did not vary significantly, which suggests these structural changes were not due to increased number of vesicles in the varicosity before it splits but rather to formation of a distinct swelling along the axon. However, no regional difference was found suggesting that the fiber/neuron interaction is similar in all the mPFC subregion examined.

### Conclusion

Investigating the local variation in the density of varicosities is a useful tool to visualize at a subcellular level plastic changes induced by functional interaction between neuronal systems and better understand the specificity of the neuronal network.
